# Boarding Schools as Disseminators of Disease

**Published:** 1895-02-16

**Authors:** 


### The Hospital

An Institutional Journal of

The Medical Sciences and Hospital Administration, with

AN EXTRA NURSING SUPPLEMENT.

Vol. XVII.—No. 438.] [Feb. 16, 1895.

### Boarding Schools as Disseminators of Diseases.

In a thickly populated country like our own one of the first cares of parents and of public bodies should be the reduction of diseases of the infectious order to a minimum. Since there is no necessity in the conditions of human life which actually compels us, or any of our children, to be the victims of infectious diseases, it is manifest that the wise parent and the right-minded public sanitarian will have this for his aim, to do all which lies in his individual power to exterminate such deadly enemies of adult and child life as diseases which are transmitted by the one only way of infection, from person to person.

Dr. Clement Dukes, physician to Rugby School, has set nothing less than this high ideal before himself; and well he fights the battle against this most deadly and destructive of all our foes. One more pamphlet, of an order as practical and high-purposed as any of its many predecessors, has been written by Dr. Dukes. Its purpose is to arraign school managers and the parents of boarding school-boys before the bar of public opinion in the interests alike of public, parents, boys, and schools. "I have this charge to bring against boarding schools," says Dr. Dukes, "that they have not exercised sufficient care in this direction, and that they have not in practice enforced the maxim of 'doing as they would be done by.'" That is not a charge to be lightly made, nor is it made lightly. Many people can testify to the statement that when parents send back their children to school after they have suffered from infectious diseases, and give the school no intimation of the fact, "great is the outcry" on the part of the school authorities. But, on the other hand, when the schools in their turn commit a similar offence, the parent is denounced as "unreasonable" who ventures to utter a single word of complaint. Summed up in a sentence, the substance of Dr. Dukes' accusation against boarding schools is this: That they disseminate infectious diseases in all directions, when they need not and ought not to do so; and that they are unreasonably indignant with parents when the latter behave to the schools exactly as the schools behave to them.

The public is principally interested in the former of these two accusations. As Dr. Dukes reminds us, the school year consists of three terms and three vacations. Boys and girls on their way to and from school, therefore, travel six times a year over the same ground, by carriage, cab, or rail. If an epidemic break out at school, the victims are sent home, as a rule, so soon as the subsidence of the acute symptoms allows of their being removed, and long before the period of infection is passed. Every convalescent who is thus sent home is a source of infection to any person with whom he may come in contact during or after his return home. Conversely, if an epidemic break out in the home, and a boy be sent to school before the period of infection is passed, he in his turn may infect any vehicle in which he may travel, and the whole school when he reaches his destination. It would seem, then, that boys going from home to school are likely to be sources of danger to a much larger number of persons than by travelling from school to their homes. But in practice it is not so, says Dr. Dukes. For, as a matter of fact, the parent is usually both so anxious about his child, and so wholesomely afraid of the anger of the school authorities, that he does not send his boy or girl back to school, as a rule, before the period of infection is well passed. Moreover, the school authorities will not admit boys who have suffered from infectious diseases without adequate medical certificates of health and freedom from infectiveness. In practice, therefore, it works out to this result, that schools are comparatively free from risks by the return of boys from their homes, whilst the homes are exposed to very great and very insidious dangers by the return of children from school.

The community is interested in this question in a dual capacity; in its capacity of parenthood, and in its capacity as a travelling public. That this mode of disseminating deadly poisons throughout the land should be promptly and effectively checked if it can be, nobody will deny. It can be checked, and that by the most simple means. All that is necessary is for schools on their part, and parents on theirs, to keep children who have been attacked by infectious diseases entirely isolated, not only until the period of convalescence, but until the period of infection is entirely passed. Most parents do this already, Dr. Dukes tells us. But most schools do exactly the opposite. Without one thought for parents or the public they pack off their infectious cases to their own homes the very first moment they are fit to travel. Now this ignorant and unsanitary barbarity must be put a stop to, and the public must put a stop to it in its own wide interests. There is a law upon the point, which makes it penal to send a person by public vehicle who is suffering from an infectious disease. But the law, principally because of the difficulty of its application, is for all practical purposes a dead letter. The public will do well to bring pressure to bear upon schools to persuade them to do in this matter as they would be done by. If persuasion cannot be made to prevail, then the law must be made sufficiently powerful to put an effective check upon so great, growing, and unnecessary an evil.

